# Whole-exome sequencing combined with postoperative data identify c.1614dup (CAMKK2) as a novel candidate monogenic obesity variant

**DOI:** 10.3389/fendo.2024.1334342

**Published:** 2024-02-26

**Authors:** Yan Wang, Chao Yang, Jun Wen, Lingling Ju, Zhengyun Ren, Tongtong Zhang, Yanjun Liu

**Affiliations:** ^1^ Center of Gastrointestinal and Minimally Invasive Surgery, Department of General Surgery, The Third People’s Hospital of Chengdu, Affiliated Hospital of Southwest Jiaotong University, Chengdu, China; ^2^ Medical Research Center, The Third People’s Hospital of Chengdu, Affiliated Hospital of Southwest Jiaotong University, Chengdu, China; ^3^ West China Second University Hospital, Sichuan University, Chengdu, China; ^4^ Institute of Biomedical Engineering, College of Medicine, Southwest Jiaotong University, Chengdu, China

**Keywords:** monogenic obesity, WES, bariatric surgery, CAMKK2, weight loss

## Abstract

Early-onset obesity is a rising health concern influenced by heredity. However, many monogenic obesity variants (MOVs) remain to be discovered due to differences in ethnicity and culture. Additionally, patients with known MOVs have shown limited weight loss after bariatric surgery, suggesting it can be used as a screening tool for new candidates. In this study, we performed whole-exome sequencing (WES) combined with postoperative data to detect candidate MOVs in a cohort of 62 early-onset obesity and 9 late-onset obesity patients. Our findings demonstrated that patients with early-onset obesity preferred a higher BMI and waist circumference (WC). We confirmed the efficacy of the method by identifying a mutation in known monogenic obesity gene, *PCSK1*, which resulted in less weight loss after surgery. 5 genes were selected for further verification, and a frameshift variant in *CAMKK2* gene: NM_001270486.1, c.1614dup, (p. Gly539Argfs*3) was identified as a novel candidate MOV. This mutation influenced the improvement of metabolism after bariatric surgery. In conclusion, our data confirm the efficacy of WES combined with postoperative data in detecting novel candidate MOVs and c.1614dup (CAMKK2) might be a promising MOV, which needs further confirmation. This study enriches the human monogenic obesity mutation database and provides a scientific basis for clinically accurate diagnosis and treatment.

## 1 Introduction

The increase in obesity presents a significant health issue as it has become a major contributor to the global occurrence of chronic diseases, including type 2 diabetes, cardiovascular diseases and some types of cancer (1, 2). Of particular concern is early-onset obesity, which refers to obesity that develops during childhood or adolescence. The prevalence of early-onset obesity is on the rise in low-income and middle-income countries, as well as many high-income countries (3). The role of genetics in the transmission of early-onset obesity and its associated health conditions is undeniable. Through genome-wide association studies (GWAS) and whole-exome sequencing (WES), numerous genetic loci linked to obesity have been identified (4, 5). However, a significant portion of patients with obesity remains unexplained by the currently identified individual genes. This implies that numerous unknown genes are yet to be discovered, and this genetic diversity is especially enhanced by differences in ethnicity and culture.

Bariatric surgery has emerged as an effective treatment for severe obesity, leading to sustained weight loss over a 10-year period and improvements in comorbidities and quality of life (6). However, it has been reported that patients carrying known MOVs experience lower weight loss after undergoing bariatric surgery (7), suggesting long-term negative effects of MOVs on patients following bariatric surgery. We therefore wondered whether this could be used to screening novel candidate MOVs.

In the current study, we employed WES in combination with weight data obtained after bariatric surgery to identify candidate MOVs that are enriched in patients with early-onset obesity. We hypothesized that candidate MOVs would be selectively enriched in the cases and filtered out in the control group. Our study successfully identified *PCSK1*, a known monogenic obesity gene, in 4 patients who experienced less weight loss after surgery, thereby confirming the validity of our method. Additionally, our study provides evidence for another candidate MOV found in the coding sequence of the *CAMKK2* gene.

These findings highlight the potential of using WES combined with post-surgical weight data to identify and characterize candidate MOVs. By expanding our understanding of the genetic factors contributing to obesity, we can gain insights into the underlying mechanisms and potentially develop more personalized and targeted approaches for the management and treatment of obesity.

## 2 Methods

### 2.1 Subjects

Obese patients (BMI >30kg/m^2^) who underwent bariatric surgery at the Gastrointestinal Minimally Invasive Center of the Third People’s Hospital of Chengdu during years of 2021 to 2022 were selected. The early-onset obesity group included subjects who were obese before 10 years old while the control group were after, according to the criteria of the Working Group for Obesity in China (WGOC) (8). Finally, a total of 62 early-onset obesity and 9 controls were included. Patient blood samples were collected, and preoperative clinical data and postoperative follow-up information were recorded. All procedures involving human subjects were approved by the Ethics Committee of The Third People’s Hospital of Chengdu. The clinical trial registration number is ChiCTR2300073353. The volunteers were provided with full information about the study, and their informed consent was obtained.

### 2.2 Whole-exome sequencing and variants analysis

Genomic DNA was isolated from peripheral blood using the Blood Genome DNA Extraction Kit (TIANGEN Biotech, Beijing, China) according to manufacturer’s protocols. Whole-exome sequencing analysis was performed using the Illumina HiSeq2500 platform (Illumina San Diego, CA, USA) with 150 bp paired-end reads. All reads were mapped against the hg38 reference genome using the Burrow–Wheeler aligner (BWA). The identification of variants was performed using a custom pipeline that utilizes the GATK Best Practices workflow (9).

Variants were filtered according to minor allele frequency (MAF) ≥ 5% in the 1000 Genomes and Exome Aggregation Consortium (ExAC) databases. Variants showed in the controls and synonymous variants, assumed to be benign or likely benign, were also excluded. Frameshift, stop gain and stoploss were considered damaging. The nonsynonymous variants were predicted to be damaging by annotation in dbNSFP database. In detail, different prediction software (SIFT, Polyphen2_HDIV, Polyphen2_HVAR, LRT, MutationTaster, MutationAssessor, FATHMM, PROVEAN, MetaSVM, MetaLR, M-CAP, CADD, fathmm-MKL) were used to assign scores for the nonsynonymous variants. Considering that some software may lack annotations for certain mutations, for those with available scores, if half or more of the software predicts damaging, the variant is considered deleterious. Then, Criteria of the American College of Medical Genetics and Genomics (ACMG) were used to determine the pathogenicity of variants, building on the automatic classification provided by VarSome (https://varsome.com/). Finally, variants for further verification were selected based on mutation category and literature data.

### 2.3 Statistical analysis

The early-onset subjects were divided into groups of non-carriers and carriers depending on whether they carried the particular variant in each comparison, e.g., non-carriers of PCSK1 mutation vs carriers of PCSK1 mutation. In the postoperative period of bariatric surgery, patients’ weight was followed up at 1 month, 3 months, 6 months, and 12 months, and the percent of weight loss was calculated. Independent *t* tests were conducted to compare the percentage of weight loss between the two groups at each time point, and the *P* values were adjusted via the FDR. Paired *t* tests were used to compare HDL, LDL, TC and TG between baseline and 12 months after surgery. To compare the percentage changes in HDL, LDL, TC and TG from baseline to 12 months post-surgery between CAMKK2 non-carriers and carriers, independent *t* tests were also performed. A value of 0.05 was considered to be statistically significant. Statistical analyses were carried out with the use of IBM SPSS Statistics, GraphPad Prism 8 and R software.

## 3 Results

### 3.1 Clinical characteristics of patients

All individuals included in the whole exome sequencing (WES) and assessments were classified as obese (BMI >30 kg/m^2^). Control subjects were selected from individuals who developed obesity after the age of 10 (n=9), while the early-onset obesity group consisted of individuals who developed obesity before the age of 10 (n=62). The clinical characteristics of both groups are summarized in **Table 1**.

**Table 1:** Clinical information of individuals with obesity.

Subject	Control (n=9)	Early-onset obesity (n=62)	*P* value
Male/Female	4/5	29/33	
Age (years)	30.00 ± 5.94	28.84 ± 8.25	
Hypertension	1	10	
Diabetes	2	13	
BMI (kg/m^2^)	33.52 ± 3.54	40.13 ± 5.95	0.002
WC (cm)	110.64 ± 8.20	121.21 ± 12.69	0.02
WHR	0.99 ± 0.04	1.01 ± 0.07	
VFL	17.89 ± 2.67	19.10 ± 1.87	
PBF (%)	42.91 ± 7.00	44.85 ± 5.78	

The results showed a significant difference in BMI and waist circumference (WC) between the control and early-onset obesity groups (*P*< 0.05), with the latter exhibiting higher BMI and WC, suggesting that early-onset obesity has a more pronounced impact on overall weight gain compared to individuals who develop obesity later in life. However, there is no significant change in waist-hip ratio (WHR), visceral fat level (VFL), and percent body fat (PBF) between them, indicating that early-onset obesity has a greater impact on weight rather than on fat distribution, potentially influenced by genetic factors that influence appetite and energy homeostasis.

### 3.2 Identification of MOVs

WES was performed for 62 early-onset obesity and 9 late-onset obesity and an average sequencing depth of 100× was obtained. Variants were selected according to mutation damaging prediction software, the American College of Medical Genetics (ACMG) guidelines (10) and literature data for further analysis. Candidate MOVs were verified by the weight loss data after bariatric surgery, as previous study reported that MOVs lead to less weight loss after bariatric surgery (7).

A total of 567 variants (variant frequency ≥5%) predicted as deleterious were identified in patients with early-onset obesity (including nonsynonymous SNVs and frameshift Indels). 11 were predicted pathogenic (P) or likely pathogenic (LP) and 60 were considered variants of uncertain significance (VUS) according to ACMG guidelines (**Supplementary Table 1**). We found 1 missense variant in the *PCSK1* gene (c.242G>A) (**Table 2**) that was already known to be associated with monogenic early-onset obesity (11, 12), which mutation led to less weight loss after bariatric surgery (7). We also found 5 genes (2 frameshift insertion, 2 frameshift deletion and 1 stop-loss) reported associating with obesity, adipogenesis or previously linked to obesity-associated traits by genome-wide association studies: *SLC25A5* (13), *AIM2* (14), *SNX16* (15), *CAMKK2* (16–18), *PDE11A* (19) (**Table 2**) and they were selected for further verification.

**Table 2:** Variants selected for further analysis.

Gene	Transcript	Variant	Protein	dbSNP	1000G	ACMG classification	ACMG tags	Carriers/Total
SLC25A5	NM_001152.5	c.450del	p. Ala150fs*64	rs759019641	.	LP	PVS1, PP5	9/62
AIM2	NM_004833.3	c.1029dup	p. *344Ileext*3	rs1557889335	0.0129792	LP	PM4, PM2, BP4	4/62
SNX16	NM_152836.3	c.1033T>C	p. *345Glnext*15	rs150053915	0.00199681	LP	PM4, PM2, BP4	4/62
CAMKK2	NM_001270486.1	c.1614dup	p. Gly539Argfs*3	NA	.	VUS	PM2	8/62
PDE11A	NM_001077197.2	c.20_21del	p. Arg7Thrfs*30	rs202117698	0.00219649	VUS	PVS1, PP5, BS2	4/62
PCSK1	NM_000439.5	c.242G>A	p. Arg81Lys	NA	.	VUS	PM2	4/62

*means change in a stop codon. NA, Not Available.

**Figure 1:**
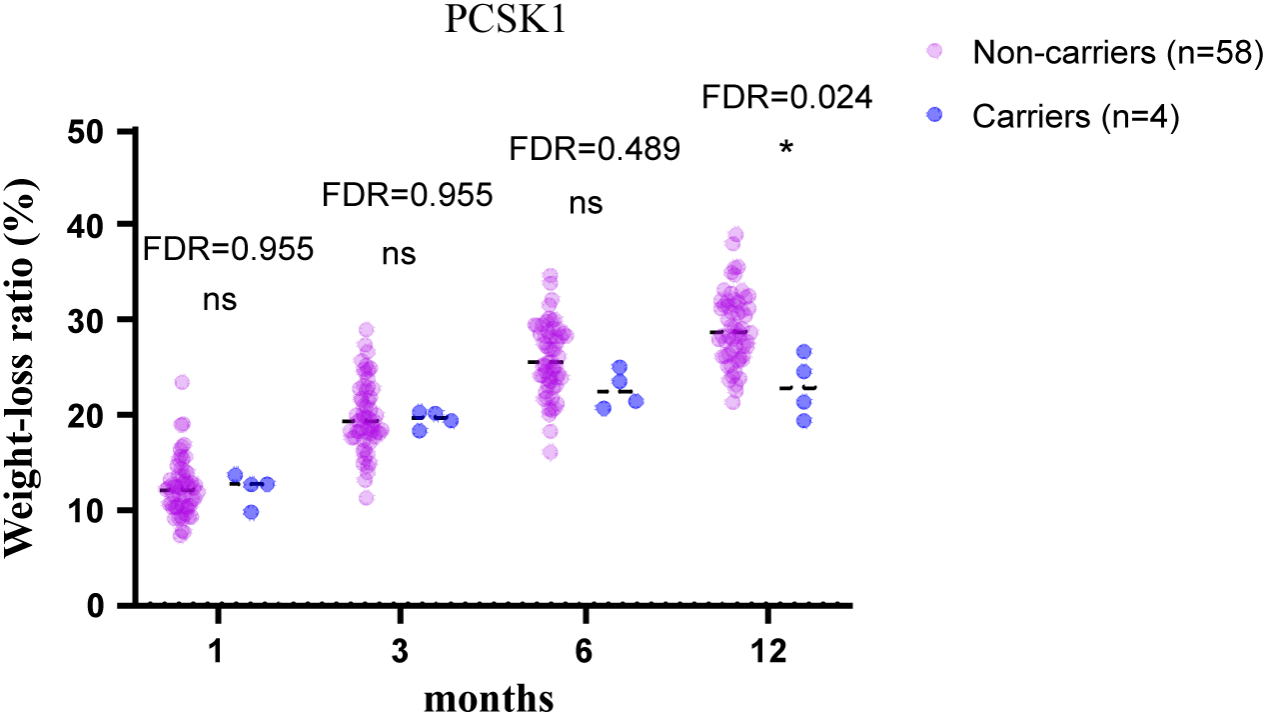
12-month weight-loss ratio for obese individuals with/without the PCSK1 mutation after bariatric surgery. PCSK1 variant led to less weight loss after bariatric surgery compared with PCSK1 non-carriers. Independent *t* tests were conducted at 1, 3, 6, 12 months, respectively, and the *P* values were adjusted via the FDR. *FDR<0.05, ns, no significance.

**Figure 2:**
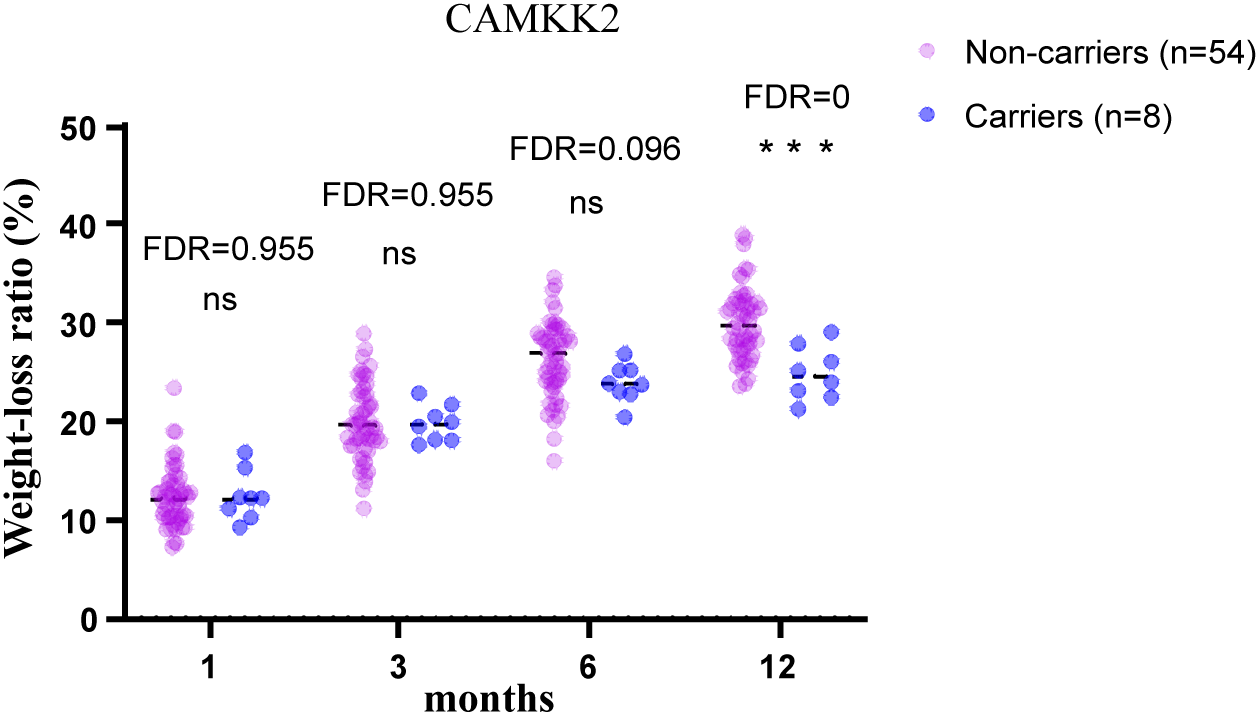
12-month postoperative weight-loss ratio in obese patients with/without CAMKK2 mutation. CAMKK2 variant carriers exhibited poorer weight loss after bariatric surgery compared with CAMKK2 non-carriers. Independent *t* tests were performed at each time point and the *P* values were adjusted via the FDR. ***FDR<0.001, ns, no significance.

To test the validity of the post-operative data to identify candidate MOVs, analysis was first performed on PCSK1 mutation carriers, and the results showed that the PCSK1 mutation resulted in less weight loss with a significant difference (FDR<0.05) (**Figure 1**), indicating that the method is effective. Therefore, to validate whether other gene variants are candidates, analysis were performed between non-carriers and carriers of each variant, including c.450del (SLC25A5), c.1029dup (AIM2), c.1033T>C (SNX16), c.1614dup (CAMKK2), c.20_21del (PDE11A). Compared to the non-carriers, CAMKK2 variant carriers exhibited poorer weight loss with a significant difference (FDR<0.001) (**Figure 2**), with no overlapping individuals with PCSK1 variant carriers, indicating that the effect of the CAMKK2 variant on weight loss is independent. AIM2 variant carriers also showed less weight loss from 1 month until 12 months after surgery, but similar with other three gene variants (c.450del (SLC25A5), c.1033T>C (SNX16), c.20_21del (PDE11A) carriers, the difference was not statistically significant compared to the non-carriers group (**Supplementary Figure 1**). In order to rule out other genetic mutations carried by CAMKK2 mutation carriers resulting in less weight loss, we took the intersection of all the mutations carried by the 8 CAMKK2 mutation carriers, and none of the mutations were screened. Therefore, we consider c.1614dup (CAMKK2) might be a promising MOV but needs further confirmation.

The calcium/calmodulin dependent protein kinase kinase 2 (CAMKK2) is a member of the serine/threonine protein kinase family and Ca(2+)/calmodulin-dependent protein kinase subfamily requiring the presence of calcium and calmodulin to be active. The c.1614dup (CAMKK2) variant involves the insertion of an A base at position 1615, which is identical to position 1614. This results in the mutation of glycine to arginine at position 539 of the CAMKK2 protein and a premature truncation from 556 amino acids to 540 amino acids. It is important to note that this mutation is predicted to cause nonsense-mediated mRNA decay (NMD), a process by which mRNAs that contain premature termination codons are degraded, indicating that even though it only reduces 16 amino acids, it is critical for the stability of this protein.

### 3.3 CAMKK2 variant influences metabolism improvement after bariatric surgery

**Figure 3:**
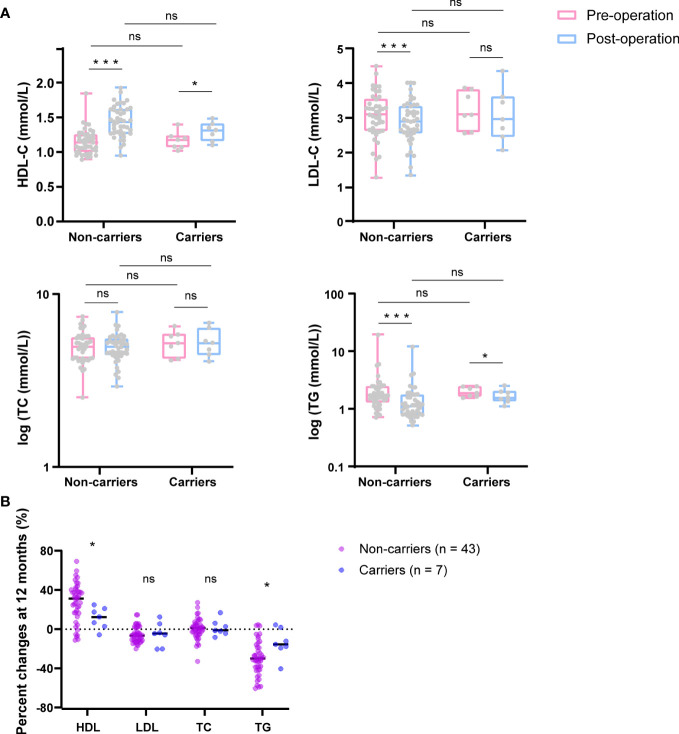
CAMKK2 variant influences metabolism improvement after bariatric surgery. **(A)** Changes in HDL, LDL, TC, and TG after bariatric surgery (12months) (blue) compared with baseline (pink) between CAMKK2 mutation non-carriers (n = 43) and carriers (n = 7). **(B)** Percentage changes from baseline to 12 months post-surgery of HDL, LDL, TC and TG. TC and TG were logarithmically transformed using GraphPad Prism 8. Compared *t* tests and independent *t* tests were performed, and the *P* values were adjusted via the FDR. *FDR<0.05, ***FDR<0.001, ns, no significance.

To evaluate the effect of c.1614dup (CAMKK2) on metabolism after bariatric surgery, we compared lipid levels between baseline and 12 months post-surgery between carriers and non-carriers of the CAMKK2 mutation (**Figure 3A**), and compared their percentage change from baseline to 12 months after surgery (**Figure 3B**). Final data were collected from 43 non-carriers and 7 carriers of the mutation due to loss of follow-up in some patients. Both groups showed a statistically significant increase in HDL cholesterol levels (mean HDL levels increased from 1.15mmol/L to 1.44mmol/L in CAMKK2 mutation non-carriers, FDR = 0, and from 1.17mmol/L to 1.30mmol/L in CAMKK2 carriers, FDR = 0.038) (**Figure 3A**), and there was a significant difference in the percentage change between the two groups (CAMKK2 non-mutation carriers 27.3%, CAMKK2 mutation carriers 11.5%, FDR = 0.046) (**Figure 3B**). LDL cholesterol was down-regulated in both groups. However, the changes between baseline and 12 months after surgery were statistically significant in CAMKK2 mutation non-carriers but not in carriers (**Figure 3A**), likely due to the limitation of the sample size. Furthermore, there was no significant difference in the percentage change between the two groups (**Figure 3B**). Meanwhile, both groups exhibited a statistically significant decrease in triglyceride (TG) levels (**Figure 3A**) and the percentage decrease was greater in individuals without the CAMKK2 mutation (29.5%) compared to those with the mutation (14%) (FDR = 0.029) (**Figure 3B**). However, no significant changes in total cholesterol (TC) levels were observed in either group (**Figure 3A**) or in percentage change between the two groups (**Figure 3B**). The results indicate that patients, regardless of whether they carry the CAMKK2 mutation or not, experience improved metabolism after undergoing bariatric surgery. However, the degree of improvement varies, with non-carriers of the CAMKK2 mutation showing significant changes in elevated levels of HDL and reduced levels of TG compared to carriers of the CAMKK2 mutation.

## 4 Discussion

Identifying the underlying causes of early-onset obesity is a challenging task due to the complex interplay of genetic and environmental factors. WES has emerged as an effective tool for detecting novel candidate genes associated with these disorders. In a study conducted by Li et al., the researchers investigated whether MOVs influence the effectiveness of bariatric surgery (7). Similarly, the long-term outcomes of bariatric surgery were evaluated in patients with bi-allelic mutations in known monogenic obesity genes such as *POMC*, *LEPR*, and *MC4R* (20). The findings from both studies suggested that carriers of monogenic obesity gene mutations exhibit less weight loss after bariatric surgery, indicating that bariatric surgery could potentially be used as a screening method for detecting MOVs following WES analysis. In the present study, WES was performed on a cohort of 62 individuals with early-onset obesity and 9 individuals with late-onset obesity. This analysis led to the identification of a known monogenic obesity gene, *PCSK*, with a mutation that was associated with less weight loss in our study. This finding further validated the effectiveness of using WES combined with postoperative data as a method for detecting candidate MOVs. Then, we selected 5 variants in genes previously reported to be associated with obesity, adipogenesis or linked to obesity-associated traits by genome-wide association studies (*SLC25A5*, *AIM2*, *SNX16*, *CAMKK2* and *PDE11A*) for further analysis to identify candidate MOVs. In combination with weight data after bariatric surgery, we finally identified c.1614dup (CAMKK2) as a novel candidate MOV.

CAMKK2 is expressed both in hypothalamus (17) and preadipocytes (16). It plays a key role in the calcium/calmodulin-dependent (CaM) kinase cascade through the phosphorylation of the downstream kinases, including CaMKI, CaMKIV and AMP-activated protein kinase (AMPK) (17, 21–23). Among these, AMPK is a key regulator of cellular energy balance, which is involved in the leptin-melanocortin signaling pathway (24). CAMKK2 null mice developed obesity, insulin resistance, and less glucose tolerance with standard chow whereas displayed a considerably smaller increase in adiposity and adipocyte size with high-fat diet compared with WT mice by regulating NPY and therefore affecting appetite (17). NPY is a known appetite regulator and its mutation led to obesity and metabolic syndrome (25). *In vitro*, inhibition or deletion of CAMKK2 in preadipocytes promoted their differentiation into mature adipocytes, which could be rescued by AMPK activation (16). These studies suggest that the CAMKK2 variant carriers identified in this study may have followed a normal diet structure instead of a high-fat diet, which surprisingly lead to weight gain. After bariatric surgery, all patients, including CAMKK2 variant carriers, are instructed to follow a specific dietary structure, which is more closely aligned with a standard diet, rather than a high-fat diet, leading to suboptimal weight loss.

Among the other genes variants that lacked statistical significance, AIM2 necessitates further validation. AIM2, a member of the IFN-inducible HIN200 protein family (26), is a cytoplasmic double-stranded DNA sensor and a tumor suppressor (27, 28). AIM2 knockout mice exhibited increased both subcutaneous and visceral adiposity compared to WT, with no differences in food intake. For the reason, AIM2 null mice showed a reduction in energy expenditure and impaired function of their brown adipose tissue. Furthermore, AIM2 knockout led to insulin resistance and glucose intolerant (14). AIM2 variants have not been reported to cause obesity yet, and in our study, a frameshift variant of AIM2 was found in 4 patients who had a family history of obesity. Although their weight loss after bariatric surgery did not reach statistical significance, it is important to note that the sample size was limited, suggesting additional studies with larger sample sizes or different populations may help to clarify whether AIM2 is a candidate monogenic obesity gene.

Furthermore, in 70 genes predicted pathology, likely pathology and variants of uncertain significance, MPP2 is predominantly expressed in the brain (Genotype-Tissue Expression), which is an established key region in the regulation of energy homeostasis and food intake. However, its role in obesity or metabolic disorders has not been reported and need to be further explored in the future.

In summary, the present investigation indicates that individuals with early-onset obesity have significantly higher BMI and waist circumstance (WC) compared to subjects with late-onset obesity. This data verified the efficiency of WES combined with postoperative data to identify candidate monogenic obesity genes and also identified c.1614dup (CAMKK2) as a promising MOV in a cohort of patients experiencing early-onset obesity. Further research with larger sample sizes is needed to confirm these findings and to determine the long-term effects of the c.1614dup (CAMKK2) variant on metabolism.

## 5 Conclusions

WES combined with weight data following bariatric surgery is an effective strategy for identifying novel candidate MOVs and c.1614dup (CAMKK2) might be a new MOV.

## Data availability statement

The datasets presented in this study can be found in online repositories. The accession number is PRJNA1041755 (https://www.ncbi.nlm.nih.gov/bioproject/PRJNA1041755).

## Ethics statement

The studies involving humans were approved by The Ethics Committee of The Third People’s Hospital of Chengdu. The studies were conducted in accordance with the local legislation and institutional requirements. Written informed consent for participation in this study was provided by the participants’ legal guardians/next of kin.

## Author contributions

YW: Investigation, Validation, Writing – original draft, Visualization, Writing – review & editing. CY: Data curation, Validation, Writing – review & editing. JW: Resources, Writing – review & editing. LJ: Investigation, Writing – review & editing. ZR: Resources, Writing – review & editing. TZ: Funding acquisition, Supervision, Writing – review & editing, Project administration. YL: Funding acquisition, Supervision, Writing – review & editing, Project administration.
