# Supplementary material for: Whole-exome sequencing combined with postoperative data identify c.1614dup (CAMKK2) as a novel candidate monogenic obesity variant
Source: Front Endocrinol (Lausanne). 2024 Feb 26;15:1334342. doi: 10.3389/fendo.2024.1334342 (PMC10925648; doi:10.3389/fendo.2024.1334342)
Supplement: Supplementary file 1 [file DataSheet_1.docx]

Supplementary Material

# Supplementary Figures


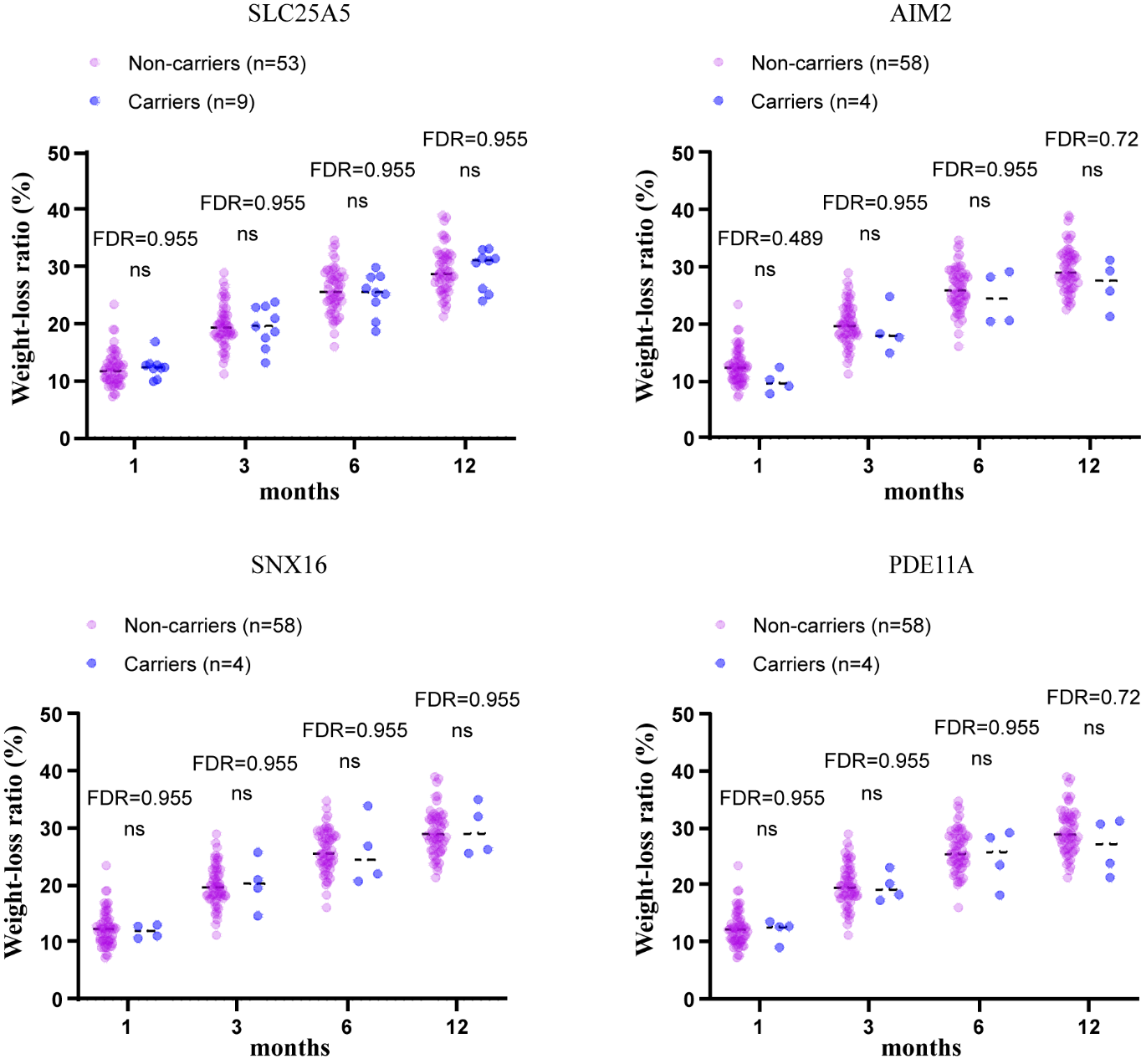


**Figure S1.** 12-month postoperative weight-loss ratio in obese patients with/ without SLC25A5, AIM2, SNX16 or PDE11A mutations. All of them showed no difference in efficiency of weight loss compared with non-carriers. Independent *t* tests were conducted at 1, 3, 6, 12 months, respectively, and the *P* values were adjusted via the FDR.

# Supplementary Tables

**Table S1.** Variants were predicted pathogenic(P), likely pathogenic(LP), or variants of uncertain significance(VUS) according to ACMG guidelines

| Gene | Mutation type | AAChange | dbSNP | 1000G | ACMG classification | ACMG tags | Carriers/Total |
| --- | --- | --- | --- | --- | --- | --- | --- |
| ZNF717 | stopgain | ZNF717:NM_001324027:exon7:c.C2348G:p.S783X | rs200767888 | . | VUS | PM2 | 13/62 |
| SH2B3 | nonsynonymous SNV | SH2B3:NM_005475:exon2:c.C724T:p.P242S | rs78894077 | 0.0127796 | VUS | PM2, BP1 | 12/62 |
| PRAMEF15 | frameshift deletion | PRAMEF15:NM_001098376:exon2:c.109delC:p.L37fs | . | . | VUS | PM2 | 11/62 |
| SEPT2 | frameshift insertion | SEPT2:NM_001349287:exon3:c.111dupT:p.Y37fs | . | . | VUS | PM2 | 10/62 |
| CTAGE4;CTAGE8 | nonsynonymous SNV | CTAGE8:NM_001278507:exon1:c.C1915T:p.P639S,CTAGE4:NM_198495:exon1:c.C1915T:p.P639S | rs201839312 | . | VUS | PM2, BP4 | 10/62 |
| OR8G2P | frameshift deletion | OR8G2P:NM_001291438:exon1:c.321delC:p.Y107fs | . | . | VUS | PM2 | 9/62 |
| BCAS3 | frameshift insertion | BCAS3:NM_001320470:exon25:c.2673dupA:p.G891fs | rs749349621 | . | VUS | PVS1, BS1 | 9/62 |
| MYO6 | frameshift insertion | MYO6:NM_001300899:exon26:c.2743dupA:p.Q914fs,MYO6:NM_004999:exon26:c.2743dupA:p.Q914fs | rs551348450 | 0.0035943 | P | PVS1, PS3 | 9/62 |
| SLC25A5 | frameshift deletion | SLC25A5:NM_001152:exon2:c.450delT:p.A150fs | rs759019641 | . | LP | PVS1, PP5 | 9/62 |
| CAMKK2 | frameshift insertion | CAMKK2:NM_001270486:exon16:c.1614dupA:p.G539fs | . | . | VUS | PM2 | 8/62 |
| PRAMEF12 | frameshift insertion | PRAMEF12:NM_001080830:exon3:c.1415_1416insCC:p.C472fs | rs199736234 | 0.009984 | VUS | PM2 | 7/62 |
| RBMXL1 | frameshift insertion | RBMXL1:NM_019610:exon2:c.1dupA:p.M1fs,RBMXL1:NM_001162536:exon3:c.1dupA:p.M1fs | rs772008437 | . | VUS | PM2 | 7/62 |
| ARIH2 | nonsynonymous SNV | ARIH2:NM_001349229:exon3:c.C287T:p.P96L,ARIH2:NM_001349228:exon4:c.C287T:p.P96L,ARIH2:NM_001349230:exon5:c.C287T:p.P96L | rs150911572 | 0.0053914 | VUS | PM2, PP2, BP4 | 7/62 |
| HGC6.3 | nonsynonymous SNV | HGC6.3:NM_001129895:exon1:c.C62T:p.S21F | rs61740140 | . | VUS | PM2, BP3 | 7/62 |
| PRAMEF33 | nonsynonymous SNV | PRAMEF33:NM_001291381:exon4:c.C1255A:p.L419I | . | . | VUS | PM2, BP3 | 6/62 |
| PRAMEF33 | nonsynonymous SNV | PRAMEF33:NM_001291381:exon4:c.C1288T:p.L430F | . | . | VUS | PM2, BP3 | 6/62 |
| PRAMEF33 | nonsynonymous SNV | PRAMEF33:NM_001291381:exon4:c.A1297G:p.I433V | . | . | VUS | PM2, BP3 | 6/62 |
| TRIM64 | frameshift deletion | TRIM64:NM_001136486:exon1:c.377delG:p.S126fs | . | . | VUS | PM2, BP3 | 6/62 |
| PDIA2 | nonsynonymous SNV | PDIA2:NM_006849:exon6:c.C857T:p.T286M | rs2685127 | . | VUS | PM2, BP1 | 6/62 |
| LOXHD1 | nonsynonymous SNV | LOXHD1:NM_001145473:exon9:c.G1316A:p.R439Q,LOXHD1:NM_001173129:exon9:c.G1316A:p.R439Q,LOXHD1:NM_001308013:exon21:c.G2978A:p.R993Q,LOXHD1:NM_001145472:exon23:c.G3266A:p.R1089Q,LOXHD1:NM_144612:exon40:c.G6413A:p.R2138Q | rs148468627 | 0.0047923 | VUS | PM3, PM2, BP1 | 6/62 |
| DNAH1 | nonsynonymous SNV | DNAH1:NM_015512:exon31:c.C4987T:p.R1663C | rs17052097 | 0.0053914 | VUS | PM2, PP3, BP1 | 6/62 |
| CSGALNACT1 | nonsynonymous SNV | CSGALNACT1:NM_001130518:exon4:c.G397A:p.V133M,CSGALNACT1:NM_018371:exon4:c.G397A:p.V133M | rs377493563 | 0.0013978 | VUS | PP3, PM2, BP1 | 6/62 |
| FREM1 | nonsynonymous SNV | FREM1:NM_144966:exon10:c.G1394C:p.G465A | rs41298151 | 0.0199681 | LP | PP3, PM2, BP1 | 6/62 |
| PRAMEF33 | nonsynonymous SNV | PRAMEF33:NM_001291381:exon3:c.C476T:p.T159I | . | . | VUS | PM2, BP3 | 5/62 |
| PRAMEF33 | nonsynonymous SNV | PRAMEF33:NM_001291381:exon3:c.T503G:p.F168C | . | . | VUS | PM2, BP3 | 5/62 |
| PRAMEF33 | nonsynonymous SNV | PRAMEF33:NM_001291381:exon4:c.C1204G:p.R402G | . | . | VUS | PM2, BP3 | 5/62 |
| PRAMEF33 | nonsynonymous SNV | PRAMEF33:NM_001291381:exon4:c.C1238A:p.P413H | rs879452055 | . | VUS | PM2, BP3 | 5/62 |
| PRAMEF33 | nonsynonymous SNV | PRAMEF33:NM_001291381:exon4:c.T1261A:p.Y421N | . | . | VUS | PM2, BP3 | 5/62 |
| PRAMEF33 | nonsynonymous SNV | PRAMEF33:NM_001291381:exon4:c.A1265G:p.K422R | . | . | VUS | PM2, BP3 | 5/62 |
| MUC6 | frameshift deletion | MUC6:NM_005961:exon31:c.4712delC:p.P1571fs | rs368342230 | . | LP | PVS1 | 5/62 |
| OR4D6 | nonsynonymous SNV | OR4D6:NM_001004708:exon1:c.C697T:p.R233W | rs76972978 | 0.0013978 | VUS | PM2, BP4 | 5/62 |
| GOLGA6L9 | nonsynonymous SNV | GOLGA6L9:NM_001291420:exon5:c.G93T:p.M31I,GOLGA6L9:NM_198181:exon6:c.G567T:p.M189I | . | . | VUS | PM2, BP3 | 5/62 |
| PMFBP1 | nonsynonymous SNV | PMFBP1:NM_031293:exon5:c.G577A:p.E193K,PMFBP1:NM_001160213:exon6:c.G142A:p.E48K | rs35370634 | . | LP | PVS1, PM2 | 5/62 |
| KIR2DL1;KIR2DL3 | stopgain | KIR2DL1:NM_014218:exon7:c.G871T:p.E291X | rs11665796 | . | VUS | PM2 | 5/62 |
| ADAMTS10 | nonsynonymous SNV | ADAMTS10:NM_030957:exon4:c.G401C:p.S134T | rs7255721 | . | VUS | PM2, BP1 | 5/62 |
| RASSF6 | frameshift insertion | RASSF6:NM_001270392:exon4:c.331dupA:p.R111fs,RASSF6:NM_001270391:exon5:c.367dupA:p.R123fs,RASSF6:NM_177532:exon5:c.367dupA:p.R123fs,RASSF6:NM_201431:exon5:c.463dupA:p.R155fs | rs568399323 | 0.0011981 | VUS | PM2 | 5/62 |
| HGC6.3 | frameshift insertion | HGC6.3:NM_001129895:exon1:c.319dupT:p.S107fs | rs879154470 | . | VUS | PM2 | 5/62 |
| MUC3A | nonsynonymous SNV | MUC3A:NM_005960:exon2:c.C1825T:p.P609S | . | . | VUS | PM2 | 5/62 |
| RASA4B | nonsynonymous SNV | RASA4B:NM_001277335:exon5:c.C425G:p.A142G | rs762105414 | . | VUS | PP3, PM2 | 5/62 |
| PRAMEF33 | nonsynonymous SNV | PRAMEF33:NM_001291381:exon4:c.G916A:p.A306T | rs879849157 | . | VUS | PM2, BP3 | 4/62 |
| S100A1 | nonsynonymous SNV | S100A1:NM_006271:exon3:c.C261G:p.N87K | rs1046256 | 0.0015974 | VUS | PM2, BP4 | 4/62 |
| AIM2 | frameshift insertion | AIM2:NM_004833:exon6:c.1029dupA:p.X344I | rs1557889335 | 0.0129792 | LP | PM4, PM2, BP4 | 4/62 |
| CUBN | nonsynonymous SNV | CUBN:NM_001081:exon16:c.C1951G:p.R651G | rs182512508 | 0.0025959 | P | PVS1, PM2, PP5 | 4/62 |
| AGAP6 | frameshift deletion | AGAP6:NM_001077665:exon8:c.1338_1351del:p.L446fs | . | 0.0017971 | VUS | PM2 | 4/62 |
| OR8G2P | nonsynonymous SNV | OR8G2P:NM_001291438:exon1:c.A326T:p.E109V | . | . | VUS | PM2 | 4/62 |
| KRTAP5-6 | nonsynonymous SNV | KRTAP5-6:NM_001012416:exon1:c.T358G:p.C120G | rs73404785 | . | VUS | PM2, BP4 | 4/62 |
| OR51S1 | nonsynonymous SNV | OR51S1:NM_001004758:exon1:c.T170A:p.I57N | rs12417164 | . | VUS | PM2, BP4 | 4/62 |
| GPRC5A | nonsynonymous SNV | GPRC5A:NM_003979:exon2:c.C370T:p.R124W | rs76634522 | 0.0077875 | VUS | PM2, BP4 | 4/62 |
| LDHB | nonsynonymous SNV | LDHB:NM_001174097:exon5:c.G473A:p.R158H,LDHB:NM_002300:exon5:c.G473A:p.R158H | rs200163319 | 0.0003994 | VUS | PP3, PM2 | 4/62 |
| B3GLCT | nonsynonymous SNV | B3GLCT:NM_194318:exon11:c.A902T:p.Y301F | rs114184584 | 0.0025959 | VUS | PP3, PM2, BP1 | 4/62 |
| RFC3 | frameshift insertion | RFC3:NM_002915:exon3:c.235dupA:p.S78fs,RFC3:NM_181558:exon3:c.235dupA:p.S78fs | rs757469602 | . | VUS | PM2 | 4/62 |
| GOLGA6L2 | frameshift deletion | GOLGA6L2:NM_001304388:exon8:c.2374delG:p.A792fs | . | . | LP | PVS1, PM2 | 4/62 |
| NPIPA7;NPIPA8 | nonsynonymous SNV | NPIPA7:NM_001282507:exon5:c.A472G:p.R158G,NPIPA8:NM_001282511:exon5:c.A472G:p.R158G,NPIPA8:NM_001349948:exon6:c.A472G:p.R158G,NPIPA8:NM_001349949:exon6:c.A472G:p.R158G | rs759672896 | . | VUS | PM2, BP4 | 4/62 |
| MPP2 | stopgain | MPP2:NM_001278370:exon1:c.G89A:p.W30X | rs184887467 | 0.0031949 | VUS | PM2, BP4 | 4/62 |
| SLC25A10 | frameshift deletion | SLC25A10:NM_001270953:exon11:c.1051delG:p.G351fs | rs141021586 | 0.0011981 | VUS | PM2 | 4/62 |
| RTTN | nonsynonymous SNV | RTTN:NM_173630:exon7:c.T805C:p.F269L | rs141156594 | 0.0073882 | VUS | PM2, BP1 | 4/62 |
| DNM2 | nonsynonymous SNV | DNM2:NM_001005360:exon6:c.C788T:p.P263L,DNM2:NM_001005361:exon6:c.C788T:p.P263L,DNM2:NM_001005362:exon6:c.C788T:p.P263L,DNM2:NM_001190716:exon6:c.C788T:p.P263L,DNM2:NM_004945:exon6:c.C788T:p.P263L | rs3745674 | 0.0021965 | VUS | PP3, PM2 | 4/62 |
| KIR3DL3 | frameshift insertion | KIR3DL3:NM_153443:exon5:c.780dupG:p.E260fs | . | . | VUS | PM2 | 4/62 |
| PDE11A | frameshift deletion | PDE11A:NM_001077197:exon2:c.20_21del:p.R7fs | rs202117698 | 0.0021965 | VUS | PVS1, PP5, BS2 | 4/62 |
| ANKRD36 | frameshift deletion | ANKRD36:NM_001164315:exon58:c.3460_3461del:p.S1154fs | rs141478865 | 0.0105831 | VUS | PM2 | 4/62 |
| PET117 | nonsynonymous SNV | PET117:NM_001164811:exon2:c.C157G:p.R53G | rs117280420 | 0.0045927 | VUS | PM2, BP4 | 4/62 |
| OR5H15 | frameshift deletion | OR5H15:NM_001005515:exon1:c.301delT:p.F101fs | rs377659479 | 0.0315495 | VUS | PM2 | 4/62 |
| ARAP2 | nonsynonymous SNV | ARAP2:NM_015230:exon25:c.G4018A:p.D1340N | rs76239807 | 0.0057907 | VUS | PM2, BP4 | 4/62 |
| PCSK1 | nonsynonymous SNV | PCSK1:NM_000439:exon2:c.G242A:p.R81K,PCSK1:NM_001177875:exon2:c.G101A:p.R34K | . | . | VUS | PM2 | 4/62 |
| HGC6.3 | nonsynonymous SNV | HGC6.3:NM_001129895:exon1:c.C281T:p.P94L | rs111332561 | . | VUS | PM2, BP3 | 4/62 |
| HGC6.3 | frameshift insertion | HGC6.3:NM_001129895:exon1:c.119_120insACAC:p.T40fs | . | . | VUS | PM2 | 4/62 |
| HLA-G | nonsynonymous SNV | HLA-G:NM_002127:exon5:c.C845T:p.T282M | rs12722482 | . | VUS | PM2, BP4 | 4/62 |
| MUC3A | frameshift insertion | MUC3A:NM_005960:exon2:c.7058_7059insAG:p.H2353fs | rs377746492 | . | LP | PVS1, PM2 | 4/62 |
| OR4F21 | frameshift insertion | OR4F21:NM_001005504:exon1:c.29dupC:p.S10fs | rs563787219 | 0.0029952 | VUS | PM2, BP3 | 4/62 |
| SNX16 | stoploss | SNX16:NM_152837:exon7:c.T946C:p.X316Q,SNX16:NM_152836:exon8:c.T1033C:p.X345Q,SNX16:NM_022133:exon9:c.T1033C:p.X345Q,SNX16:NM_001348189:exon10:c.T946C:p.X316Q | rs150053915 | 0.0019968 | LP | PM4, PM2, BP4 | 4/62 |
| KIAA1161 | frameshift insertion | KIAA1161:NM_020702:exon2:c.40dupC:p.R14fs | rs761312002 | . | LP | PVS1, PM2, BP6 | 4/62 |
